# Reproduction of parasitic mites *Varroa destructor* in original and new honeybee hosts

**DOI:** 10.1002/ece3.3802

**Published:** 2018-01-22

**Authors:** Zheguang Lin, Yao Qin, Paul Page, Shuai Wang, Li Li, Zhengsheng Wen, Fuliang Hu, Peter Neumann, Huoqing Zheng, Vincent Dietemann

**Affiliations:** ^1^ College of Animal Sciences Zhejiang University Hangzhou China; ^2^ Agroscope Swiss Bee Research Center Bern Switzerland; ^3^ Vetsuisse Faculty Institute of Bee Health University of Bern Bern Switzerland; ^4^ Plant Bioactive Compound Laboratory Faculty of Agriculture Chiang Mai University Chiang Mai Thailand

**Keywords:** *Apis cerana*, *Apis mellifera*, host–parasite coevolution, parasite reproduction, *Varroa destructor*

## Abstract

The ectoparasitic mite, *Varroa destructor*, shifted host from the eastern honeybee, *Apis cerana*, to the western honeybee, *Apis mellifera*. Whereas the original host survives infestations by this parasite, they are lethal to colonies of its new host. Here, we investigated a population of *A. cerana* naturally infested by the *V. destructor* Korea haplotype that gave rise to the globally invasive mite lineage. Our aim was to better characterize traits that allow for the survival of the original host to infestations by this particular mite haplotype. A known major trait of resistance is the lack of mite reproduction on worker brood in *A. cerana*. We show that this trait is neither due to a lack of host attractiveness nor of reproduction initiation by the parasite. However, successful mite reproduction was prevented by abnormal host development. Adult *A. cerana* workers recognized this state and removed hosts and parasites, which greatly affected the fitness of the parasite. These results confirm and complete previous observations of brood susceptibility to infestation in other honeybee host populations, provide new insights into the coevolution between hosts and parasites in this system, and may contribute to mitigating the large‐scale colony losses of *A. mellifera* due to *V. destructor*.

## 1 INTRODUCTION

In an era of globalization, international trade purposely or unintentionally provides opportunities for the translocation of parasites beyond natural barriers (Hulme, 2009; Meyerson & Mooney, 2007; Perrings, Dehnen‐Schmutz, Touza, & Williamson, 2005), creating opportunities to identify the processes of coevolution following host shifts (Antonovics, Hood, & Partain, 2002; Woolhouse, Haydon, & Antia, 2005). The conditions for such a shift were provided to the ectoparasitic mite *Varroa destructor* when colonies of the western honeybees, *Apis mellifera*, were introduced into Asia, in the distribution range of the original host of this parasite, the eastern honeybees, *Apis cerana* (Rath, 1999; Rosenkranz, Aumeier, & Ziegelmann, 2010). Both hosts and parasites show high genetic diversity in their natural range (Anderson & Trueman, 2000; Beaurepaire et al., 2015; Navajas et al., 2010; Warrit, Smith, & Lekprayoon, 2006). Several *Varroa* haplotypes shifted host (Anderson & Trueman, 2000; Roberts, Anderson, & Tay, 2015), but only a lineage of the Korean haplotype of *V. destructor* rapidly spread to reach a long‐lasting and near global distribution (Matheson, 1995; Neumann & Carreck, 2010). Its ubiquity exposes the invasive lineage to diverse populations of original and new hosts, providing several comparison points to investigate the range of host–parasite coevolution processes at play in the interaction between *Apis* spp. and *Varroa* spp.

The ectoparasitic mite *V. destructor* parasitizes both immature and adult honeybees. It feeds on the hemolymph of its hosts and reproduces on immature honeybees that develop in capped brood cells (Rosenkranz et al., 2010). This mite has been acknowledged as the most severe biotic threat to apiculture with *A. mellifera* in the last decades (Dietemann et al., 2012; Nazzi & Le Conte, 2016; Neumann & Carreck, 2010). *V. destructor* parasitism impacts host physiology (Amdam, Hartfelder, Norberg, Hagen, & Omholt, 2004; Bowen‐Walker & Gunn, 2001) as well as immune functions, leading to the outbreak of infectious diseases (Di Prisco et al., 2011; Yang & Cox‐Foster, 2005). Without acaricide treatment, infested *A. mellifera* colonies die within 6 months to 2 years (Korpela, Aarhus, Fries, & Hansen, 1992; Le Conte, Ellis, & Ritter, 2010). In contrast, infestation rates in *A. cerana* are low and colonies are able to survive without human intervention (Huang, 2012; Rosenkranz et al., 2010). Identifying the traits on which this resistance is based is not only of interest to better understand the mechanisms underlying host–parasite coevolution, but also has important applications toward a better control of this parasite and to guarantee the maintenance of the ecological and agro‐economic services provided by *A. mellifera* (Dietemann et al., 2012; Rosenkranz et al., 2010).

In *A. cerana*, a major trait of resistance against *Varroa* mites is the almost exclusive reproduction of *V. destructor* foundresses on the seasonally produced drone brood (Boecking, Rath, & Drescher, 1993; Boot et al., 1997; Huang, 2012; Koeniger, Koeniger, & Wijayagunasekara, 1981; Koeniger, Koeniger, & Delfinado‐Baker, 1983; Rosenkranz, Tewarson, Singh, & Engels, 1993; Tewarson, Singh, & Engels, 1992; but see De Jong, 1988 and Boot et al., 1999 for rare exceptions). In the new host, *A. mellifera*, mite reproduction also occurs on worker brood, which is accessible during several months of the year, allowing mite populations to proliferate exponentially. Infestation rates can thus reach damage thresholds and ultimately result in colony losses (Boot et al., 1997, 1999; Huang, 2012; Koeniger et al., 1983; Rosenkranz et al., 2010). The importance of the ability to reproduce on worker brood in virulence of this parasite is supported by the few cases of resistant populations of *A. mellifera*. Colonies of these populations show reduced *V. destructor* reproductive output on worker brood (Locke, Le Conte, Crauser, & Fries, 2012; Strauss et al., 2016). The mechanisms preventing or considerably restricting this reproduction have not yet been fully elucidated.

To improve our understanding of *V. destructor* resistance and in particular the traits that hinder mite reproduction in worker brood of the invasive lineage's original host, we chose a Chinese population of *A. cerana* parasitized by the Korean haplotype of *V. destructor* (Navajas et al., 2010; Zhou et al., 2004). We investigated several stages in the host–parasite interaction at which parasite reproduction could fail on worker brood. The first step for a parasite to be able to reproduce and acquire fitness is to find a host. In *A. mellifera* colonies, *V. destructor* females enter cells in which worker larvae develop just before the adult host workers seal the cells with a wax cap, ahead of pupation (De Guzman, Rinderer, & Frake, 2007; Guzmán‐Novoa, Vandame, & Arechavaleta, 1999). Here, we verified whether *A. cerana* worker larvae at this stage are attractive to the mite. Indeed, variation in attractiveness of larvae for the parasite has been observed in resistant *A. mellifera* lineages (Nazzi & Le Conte, 2016) and it could well be that *A. cerana* worker larvae do not produce the kairomones used by *V. destructor* for host finding. Based on the rare observations of *Varroa* spp. reproduction in *A. cerana* worker brood (Boot et al., 1999; De Jong, 1988), we expected this brood type to be attractive to the parasite and that reproduction would fail at a later stage. Once the host is found, initiation and completion of reproduction are required for the parasite to successfully exploit its host. We thus tested whether these steps also occur in *A. cerana* worker brood using experimental infestations. Infested brood was reared in both the absence and presence of workers to investigate their roles in determining mite reproductive success. All experiments were also performed in *A. mellifera* in order to compare the output of host–parasite interactions in a newly established relationship with the original coevolved system. Recently, we showed that susceptibility of worker brood in several Thai *A. cerana* populations was higher than that of the new host *A. mellifera* and that this could trigger a higher hygienic reaction in adult workers, thereby interrupting parasite multiplication (Page et al., 2016). We expected this phenomenon, coined social apoptosis, to also be expressed in the original host population of the Korean lineage of *V. destructor*. We here show the existence of this trait in a Chinese population, indicating its widespread occurrence in *A. cerana*. We also complete the previous study by investigating the effect of high brood susceptibility on parasite reproduction. Our results support the idea that this trait is a major determinant of the resistance to the invasive lineage of *V. destructor* of its original host, *A. cerana*.

## 2 MATERIALS AND METHODS

### 2.1 Honeybee colonies

From 2013 to 2015, experiments were performed in spring and autumn with *A. m. ligustica* and *A. c. cerana* colonies at an apiary at Zhejiang University, Hangzhou, China (120°05′25″E, 30°18′22″N). The colonies were queenright and kept in Langstroth hives, had ample honey and pollen stores, and displayed no obvious clinical symptoms of any diseases. All of the *A. mellifera* colonies were routinely treated against ectoparasitic mites using fluvalinate strips, 2–3 months prior to the experiment. Five additional *A. mellifera* colonies that had not been treated in at least 5 months were used as *V. destructor* mite donors. Thirty mites harvested from these colonies were confirmed to belong to the Korean haplotype using standard methods (Dietemann et al., 2013) by comparing mitochondrial DNA sequences to references deposited in GenBank (*V. destructor* Cox‐1 gene 458 bp fragment, accession number AF106899.1).

### 2.2 Attractiveness of *A. mellifera* and *A. cerana* worker brood for *V. destructor*

Brood combs containing several hundred 5th instar worker larvae were taken from six *A. mellifera* and six *A. cerana* colonies. The positions of these cells on the combs were mapped using transparent sheets (Dietemann et al., 2013). Then, one comb of each species was introduced simultaneously into one of three *V. destructor* donor colonies, thereby enabling mite infestations prior to cell capping by workers (*A. mellifera* workers are capable of capping *A. cerana* brood cells). The combs were withdrawn from the colonies 6 hr after their introduction in order to maximize the number of capped cells to investigate while reducing the opportunities for adult workers to hygienically remove infested brood (Page et al., 2016). Freshly capped cells were identified using the transparent sheets and opened to remove the larvae with tweezers. The presence or absence of *V. destructor* mites in these cells was then reported.

### 2.3 Reproductive success of *V. destructor* on worker brood and effect of infestation on brood development

To determine whether *V. destructor* mites initiate and complete reproduction on worker brood of *A. cerana* and to investigate the effect of infestation on the development of this brood, we experimentally infested freshly sealed larvae of *A. cerana*. As *V. destructor* is able to reproduce on worker brood of its new host, experimental infestations of *A. mellifera* worker brood served as positive controls. Infested brood was reared in the absence and presence of adult workers to determine how hygienic removal affects mite reproductive output (Harris, Danka, & Villa, 2010; Page et al., 2016).

#### 2.3.1 Mite collection

Two days prior to the experiments, adult female *V. destructor* mites were collected from worker or drone brood cells of *A. mellifera* colonies (*n* = 5). Batches of 30 mites were kept on 15 *A. mellifera* nurse workers of the same colonies to mimic the nonreproductive phase of the mite life cycle on adult hosts (Rosenkranz et al., 2010). This standardized the physiological status of the mites collected. We carefully screened the adult workers used as mite carriers and only uninfested individuals were used, thereby excluding the inadvertent use of mites that did not originate from the brood combs selected. Cages were provisioned with honey and kept in an incubator at 30°C under 65% RH (Williams et al., 2013).

#### 2.3.2 Experimental infestations with *V. destructor*

The experimental infestation method for both *A. mellifera* and *A. cerana* brood cells implemented in this experiment is described in Dietemann et al. (2013). In brief, brood cells sealed within the last 6 hr were identified by mapping on transparent sheets. During this period, the signals triggering reproduction in *V. destructor* are present in most cells (Frey, Odemer, Blum, & Rosenkranz, 2013) and allow a valid assessment of mite (foundress) reproductive capacity. The cell caps were lifted with a sterile blade, and a mite obtained from the caged workers (Section 2.3.1) was introduced with a paintbrush into each cell through the hole created. The wax caps were then pushed back down and resealed to the cell walls with the help of warm blade. Exactly the same manipulation, merely in the absence of a *V. destructor* mite, was performed on additional cells (thereafter designated as uninfested) of each tested colony. The individuals in these cells constituted controls for the effect of cell opening required for artificial infestation on brood development. Six to 49 larvae were infested per colony (*N* = 22 for *A. mellifera* and *N* = 22 for *A. cerana*). In *A. mellifera*, a total of 329 larvae were infested and 341 were left uninfested. In *A. cerana*, 257 larvae were infested and 253 left uninfested. The portion of comb containing the infested and uninfested cells was finally cut out and suspended vertically in an incubator (34.5°C, 70% RH; Crailsheim et al., 2013).

One day before the expected adult emergence (after 11 and 10 days for *A. mellifera* and *A. cerana*, respectively), the infested and uninfested cells were opened. The development of the honeybee brood was categorized in successive stages: larva, prepupa, white‐eyed pupa, pink‐eyed pupa, purple‐eyed pupa, gray wings, gray thorax, or gray abdomen (Human et al., 2013). The last three stages were typical for those observed in uninfested individuals. They were considered as representing normal development for pre‐emergence individuals under our experimental conditions. The presence of brood at earlier stages was considered as evidence of delayed development. In some individuals, the presence of abscesses, of dark coloration or of a decomposed state indicated that they were dead. Individuals with delayed development and dead individuals were considered as showing abnormal development.

Reproductive parameters of *V. destructor* foundresses were reported for each host species. These parameters included fertility (the percentage per host colony of foundresses that produced offspring), fecundity (number of offspring produced per foundress), the developmental stage of offspring (egg, protonymph, deutonymph female, adult male, and adult female, Dietemann et al., 2013), and reproductive success. The latter was defined as the number of mature daughter mites reared in the presence of a male (Dietemann et al., 2013). We also measured the proportion of foundress mites with reproductive success per colony.

The maximal reproductive potential of foundress mites was assessed using hosts that had reached pre‐emergence stages and excluding hosts that did not complete their development normally. The latter were, however, also considered to obtain the overall reproductive output of *V. destructor* in each species. Only foundresses that produced offspring were considered to quantify the average number of offspring of different developmental stages, the average fecundity, and the average reproductive success. Infested cells from which the foundress had escaped were counted, but discarded from the sample to evaluate reproductive parameters. Control cells that were not experimentally infested but turned out to be naturally infested and experimentally infested cells with multiple infestations were not considered in the data analyses.

### 2.4 Effect of adult workers on the reproduction of *V. destructor* in worker brood of *A. cerana* and *A. mellifera*

In order to determine the combined effects of worker brood and adults on the reproductive success of *V. destructor*, we placed infested worker brood (see Section 2.3.2) back into their original colonies. For this, we used five of the 22 colonies of each honeybee species used previously. At least 1 week before the observation started, four frames fully covered with workers of each test colonies, together with their queen, had been introduced into observation hives. Experimental infestation of 10 to 15 cells located on one of the four combs of each colony was performed following the method described above (Section 2.3.2). Two of the five *V. destructor* donor colonies (see Section 2.3.1) were used as mite supplies for these infestations. The same number of 10 to 15 sham‐treated cells was attributed to the uninfested control group of each colony. After infestation or sham treatment, the combs were returned into their colonies. The presence of test and control brood was monitored every 24 hr through the glass sides of observation hives to minimize disturbance to the colonies. Prior to this experiment, we showed that the hygienic response of these colonies to freeze‐killed brood was similar in observation hives and in larger hive units to exclude a bias of colony size and hive architecture on this behavior. Over 85% of the frozen brood was removed within 48 hr in both hive types and colonies, thus showing comparable hygienic abilities irrespective of hive type and number of individuals in the colonial units (Lin et al., 2016).

Cells in which brood had been removed were regarded as hygienically targeted. We deduced the developmental stage of targeted brood at the day of removal by comparison with the development of uninfested reference brood. This reference was established by monitoring development of uninfested workers at 1‐day intervals (Table S1, Figure S1). One day prior to the expected emergence of the imago, the cells of which the content had not been removed by adult workers were opened to determine the developmental stage of the brood and its infestation status (see Section 2.3.2). The cells from which the foundress mites escaped were counted, but discarded from the analysis of *V. destructor* reproductive parameters.

### 2.5 Statistics

The Lilliefors test showed deviation from normal distribution of errors for the total number of *V. destructor* offspring, the number of offspring at each developmental stage and the number of presumably mated daughter mites. These reproductive parameters were thus compared between *A. mellifera* and *A. cerana* with generalized linear mixed models (GLMMs, package lme4 of R v. 1.1‐14, Bates, Maechler, Bolker, & Walker, 2014). In this analysis, honeybee species was considered as fixed factor and colony identity as random factor nested within species. The function glmer of lme4 was used for modeling. As the reproductive parameters compared were count data, we used a Poisson error distribution for this model and verified the absence of overdispersion with the function dispersion_glmer of package blmeco (https://stat.ethz.ch/pipermail/r-sig-mixed-models/2011q1/015392.html). In case of overdispersion, the model was rerun with a negative binomial distribution of errors. Bonferroni's correction was applied to reduce the probability of Type I errors following multiple comparisons.

Transformation did not prevent significant deviations from normality of the error terms for the following parameters: proportions of (i) brood showing abnormal development in the absence of adult workers in infested and (ii) uninfested brood, (iii) reproductive *V. destructor* foundresses (a measure of foundress fertility), (iv) cases in which a son and at least one mature daughter were produced and (v) *V. destructor* foundresses missing from the experimentally infested brood. Generalized linear models in R (glm function) were thus performed to determine if host species significantly affected these parameters. Binomial error distribution was used given the proportional nature of the data. When necessary, that is, when residual deviance was not in the range of degrees of freedom, quasibinomial error distributions were used to account for overdispersion. A Bonferroni correction was applied to interpret the output of models for iii and iv since the data were collected from the same cells.

To compare the frequency of brood removal by workers of each species in the observation hives, we used Kaplan‐Meier plots and log‐rank (Mantel‐Cox) tests. Bonferroni's correction was applied to reduce the probability of Type I errors following multiple comparisons. Nonparametric bivariate correlation (Spearman's rank correlation coefficient) tests were performed with SPSS Statistics 22 to quantify the strength of association between brood stage at which infested brood seemed arrested in the absence of adult workers and inferred developmental stage at removal.

## 3 RESULTS

### 3.1 Attractiveness of *A. cerana* and *A. mellifera* worker larvae for *V. destructor*

**Figure 1:**
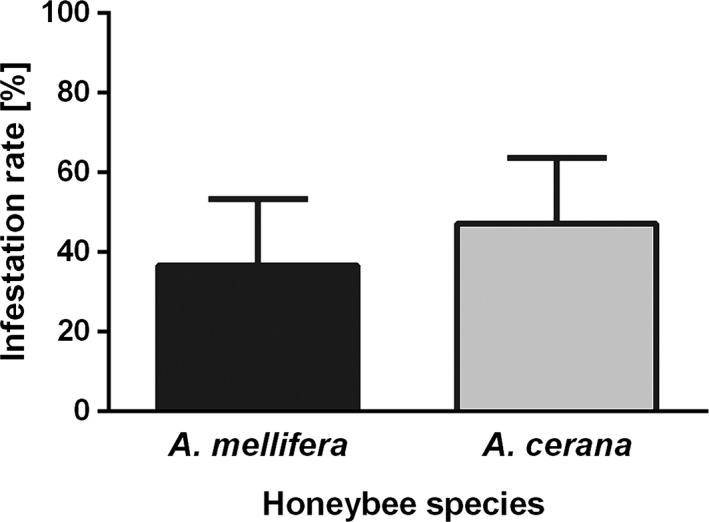
*Varroa destructor* infestation rate of freshly sealed worker brood of *Apis mellifera* and *Apis cerana*. The occurrence of mites in both test groups shows that worker larvae of *A. cerana* and of *A. mellifera* are attractive for *V. destructor*. Values are means ± *SD*

The number of freshly sealed worker brood cells available to *V. destructor* for infestation were 72.2 ± 29.6 (mean ± *SD*) per comb for *A. mellifera* and 51.0 ± 25.6 for *A. cerana*. The infestation rates of these cells were 36.7 ± 16.7% and 47.2 ± 16.4% for *A. mellifera* and *A. cerana*, respectively (Figure 1).

### 3.2 Effect of *V. destructor* infestation on development of *A. cerana* and *A. mellifera* worker brood

**Figure 2:**
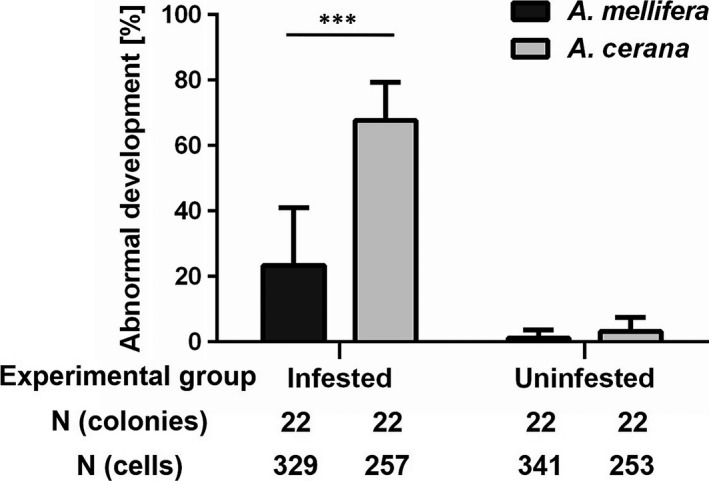
Percentage of uninfested and of *Varroa destructor* infested worker brood showing abnormal development in *Apis mellifera* and *Apis cerana*. Values are means ± *SD*. ***p < .001 for the effect of species on proportion of abnormally developed brood in a generalized linear model

In the absence of adult workers, the percentage of uninfested worker brood showing abnormal development was low and not significantly affected by host species (*z* = −1.869, *p *=* *.062; Figure 2; Table S2). As overdispersion was detected for the model for infested brood, a model with quasibinomial dispersion was used. In contrast to uninfested brood, host species significantly affected development (*t* = −7.763, *p *<* *.001; Table S2). Abnormally developed brood occurred more frequently in *A. cerana* than in *A. mellifera* (Figure 2).

### 3.3 Effect of *A. cerana* and *A. mellifera* worker brood development on *V. destructor* reproduction

**Figure 3:**
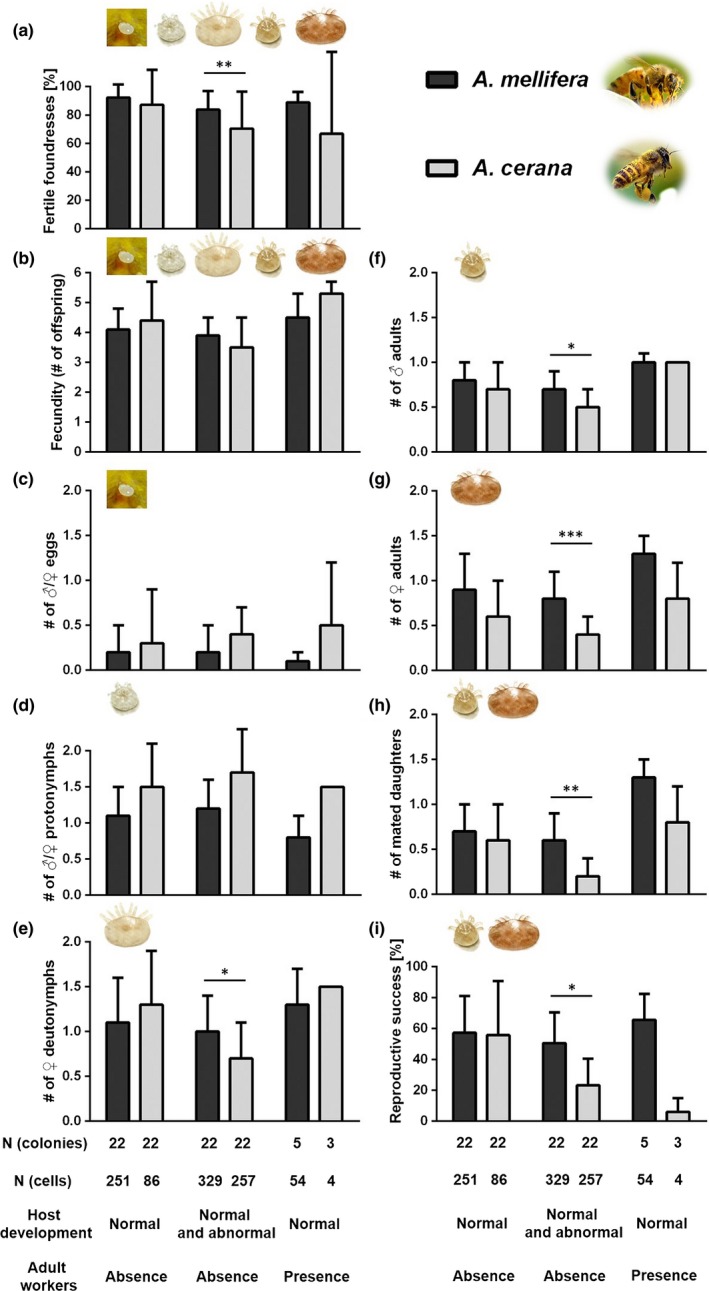
Reproductive output of *Varroa destructor* foundresses on *Apis mellifera* and *Apis cerana* worker brood. In the absence of workers, output achieved on normally developed brood reflects maximal reproductive potential of foundresses. Output reached on brood showing normal and abnormal development reflects overall reproductive potential. In the presence of workers, effective reproductive potential is assessed. Pictures above each graph show the mite offspring developmental stages and their combinations used for assessing the various reproductive parameters. Values are means ± *SD* for colonies. *p <* *.05; **p <* *.01; ***p < .001 for the effect of species on each parameter in a generalized linear mixed model, after Bonferroni's correction

In the absence of adults, *V. destructor* produced offspring on the worker brood of both species. Host species did not significantly affect the fertility and fecundity of mites reproducing on normally developing brood (*z* = 1.367, *p *=* *.172; *z* = 0.609, *p *=* *.542, respectively; Figure 3a,b; Tables S3, S4). Neither did species significantly affect the number of offspring at various developmental stages (Figure 3c–g; Table S4). Accordingly, species did not affect the number of mated daughters per foundress and the percentage of foundresses with at least one mated daughter (*z* = −0.993, *p* = .32 and *t* = 0.49, *p *=* *.62, respectively; Figure 3h,i; Tables S3, S4).

When reproduction on both normally developed brood and on brood showing abnormal development was considered, fertility but not fecundity of *V. destructor* foundresses was significantly affected by host species (*t* = −2.784, *p *=* *.008 and *z* = −1.912, *p *=* *.056, respectively; Figure 3a,b; Tables S3, S4). Except for the number of eggs and protonymphs, host species significantly affected the age distribution of mite offspring 1 day before imago emergence (Table S4). A lower number of female deutonymphs, adult males, and adult daughters were observed in *A. cerana* (Figure 3e–g). In contrast to the situation in which only normally developed brood was considered, the number of mated daughters per foundress and the percentage of foundresses with at least one mated daughter were both significantly affected by host species (*z* = −4.813, *p* < .0001, *t* = −4.778, *p *<* *.0001, respectively; Tables S3, S4). They were inferior in *A. cerana* compared to *A. mellifera* (Figure 3h,i).

### 3.4 Effect of adult workers of *A. cerana* and *A. mellifera* on *V. destructor* reproduction

**Figure 4:**
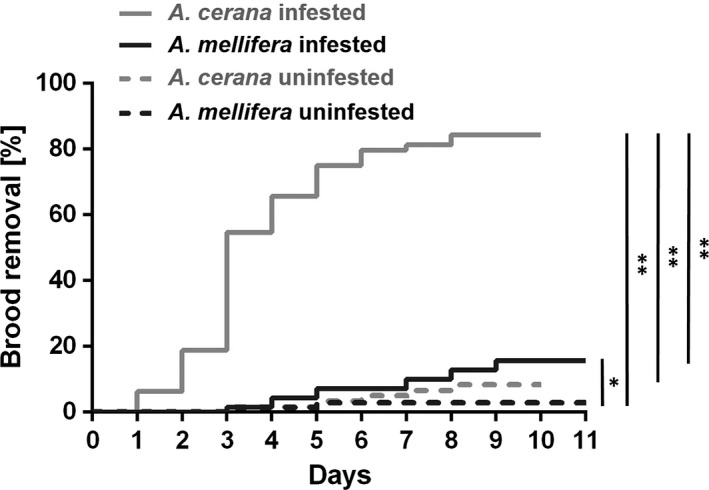
Kaplan‐Meier curves showing removal of *Varroa destructor* infested brood by adult workers of *Apis cerana* and *Apis mellifera*. Log‐rank test: *p <* *.05; **p <* *.01 after Bonferroni correction

*A. mellifera* infested brood was significantly more frequently removed by adult workers than uninfested brood (log‐rank test, χ^2^ = 6.683, *p *=* *.010; Figure 4) and the same held true for *A. cerana* (log‐rank test, χ^2^ = 81.309, *p *<* *.001; Figure 4). Frequency of removal of infested brood was significantly higher in *A. cerana* than in *A. mellifera* (log‐rank test, χ^2^ = 46.221, *p *<* *.001; Figure 4).

The generalized linear model showed that the factor host species did not affect the proportions of missing mites significantly, but that the presence of workers and the interaction between species and presence of workers did (Table S5). This indicates that the presence of workers influenced the proportion of missing mites differently between species. Due to the singularity of the contrast matrix, no pairwise tests could be performed. However, it was obvious that the presence of workers in the three *A. cerana* colonies in which experimentally infested brood remained 1 day before imago emergence was associated with the highest proportion of missing mites (83.3 ± 23.8% of the cells). This percentage was higher than in the absence of adult *A. cerana* workers (5.67 ± 8.8%). This pattern was not observed for *A. mellifera*, in which few foundresses were missing both in the presence and in the absence of adults (1.5 ± 3.4% and 4.1 ± 3.9%, respectively).

**Figure 5:**
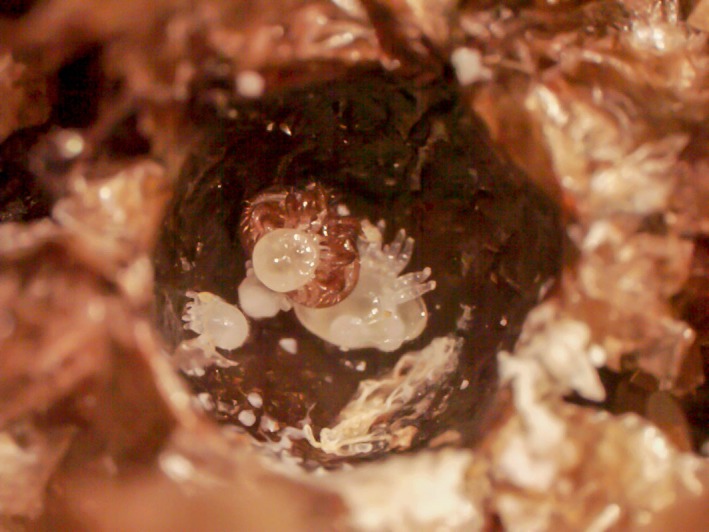
*Varroa destructor* foundress and offspring in an experimentally infested *Apis cerana* worker brood cell that escaped hygienic removal by adult worker bees. The cell was opened, and the honeybee pupa removed 1 day before expected completion of development. *V. destructor* offspring included an adult male, a deutonymph female, two protonymphs, and one egg

Foundresses parasitizing brood that was not removed by adult workers 1 day before emergence produced offspring in both *A. cerana* (Figure 5) and *A. mellifera*. Their fertility and fecundity did not significantly vary depending on the host species (*t* = 0.003, *p *=* *.998 and *z* = 0.609, *p* = .542, respectively; Figure 3a,b; Tables S3,S4). Honeybee species did not significantly affect the number of offspring at each developmental stage (Figure 3c–g; Table S4). In *A. cerana*, three foundresses yielded one presumably mated daughter each. The number of mated daughters per foundress was not significantly affected by host species (*z* = −0.607, *p *=* *.544; Figure 3h; Table S4). Because of the differential brood removal rate, host species affected the percentage of foundresses that yielded at least one mated daughter, but not significantly so (*t* = 0.041, *p *=* *.967; Table S3). This percentage was inferior in *A. cerana* (Figure 3i). The absence of significant differences in the presence of workers is likely due to the low number of infested cells that escaped hygienic removal in *A. cerana*.

### 3.5 Association between brood development and removal by adult workers

**Figure 6:**
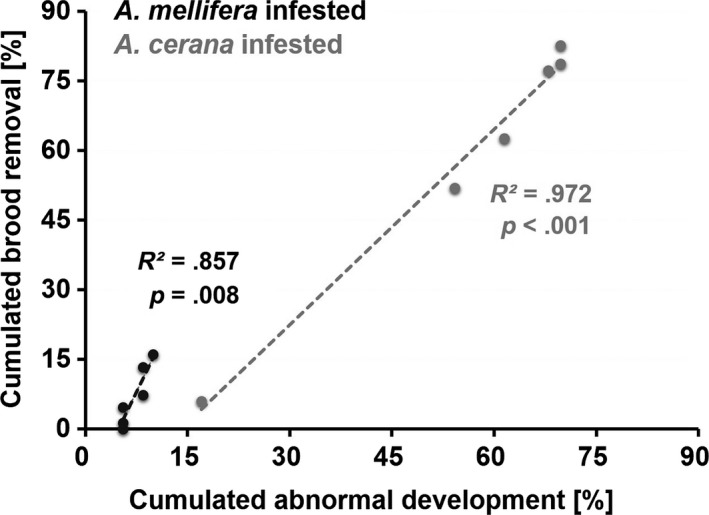
Correlations between abnormally developed infested brood and brood removal by adult worker bees in *Apis mellifera* and *Apis cerana*. The cumulated percentage of brood development in the absence of adult workers is represented on the *x*‐axis; the *y*‐axis shows the cumulated percentage of brood removal by adult workers. In each species, the dots represent the six consecutive developmental stages from lower left to upper right: larva, prepupa, white‐eyed pupa, pink‐eyed pupa, purple‐eyed pupa, and pre‐emergence adult

In *A. cerana*, prepupal and purple‐eyed stages were represented in the majority of brood showing abnormal development (75.5 ± 5.3%), most of which was arrested at the prepupal stage (53.2 ± 12.8%). In line with this pattern, the majority (74.0 ± 1.3%) of the brood removal by adult workers targeted prepupal to purple‐eyed stages (days 2–7; Figure 4; Table S1), with the most targeted (30.7 ± 11.6%) being the prepupal stage (days 2–3; Figure 4; Table S1). In *A. mellifera*, abnormal brood development and brood removal were less frequent, but also appeared to be linked. In both host species, the cumulated percentage of brood removal by adult workers was significantly correlated to the cumulated percentage of individuals showing developmental delay in the absence of adult workers (nonparametric bivariate correlation test, *A. cerana*:* R* ^2^ = .972, *p *<* *.001; *A. mellifera*:* R* ^2^ = .857, *p *=* *.008; Figure 6, Table S1). For uninfested brood, this correlation was not significant (*A. cerana*:* R* ^2^ = .501, *p *=* *.115; *A. mellifera*:* R* ^2^ = .613, *p *=* *.066). In *A. cerana*, the infested brood that had not been removed 1 day prior to imago emergence (*N* = 2 in each of 5 colonies) was at a similar developmental stage than uninfested brood.

## 4 DISCUSSION

The data clearly show that the commonly reported absence of *V. destructor* infestation in worker brood of *A. cerana* is neither due to its lack of attractiveness for the parasite nor to the absence of mite reproduction. When worker brood developed normally, the reproductive output of mite foundresses did not differ between *A. cerana* and *A. mellifera*. However, when compared to *A. mellifera,* the development of a larger proportion of *A. cerana* worker brood was negatively affected by infestation. The stages at which brood development seemed arrested corresponded to the timing of brood removal by adult workers. Both abnormal development and removal of infested worker brood negatively affected the reproductive success of *V. destructor* foundresses in *A. cerana*. Despite attraction to host larvae and initiation of reproduction, successful production of mite offspring was thus rare, but remained occasionally possible. Building on previous knowledge (Page et al., 2016), this result provides new insights into the traits providing resistance or leading to susceptibility to the invasive Korean haplotype of *V. destructor*.

### 4.1 The ability of *V. destructor* to infest and reproduce on *A. cerana* worker brood

Our data show that the commonly reported absence of the Korean haplotype of *V. destructor* infestation in worker brood of its original *A. cerana* host is not due to its lack of attractiveness for the parasite. Worker brood of *A. cerana* was infested naturally to a similar degree to that of *A. mellifera* (Figure 1). Not only were *V. destructor* foundresses attracted to *A. cerana* worker larvae, they also initiated reproduction. In the absence of adult workers that could bias the measure of mite reproductive output via hygienic behavior, all offspring stages were represented equally on both host species when brood developed normally (Figure 3). Accordingly, the maximum reproductive potential of the foundresses did not differ between the honeybee species. At the population level, effective reproductive success, however, depends on the proportion of hosts successfully completing development.

### 4.2 The effect of brood susceptibility on *V. destructor* fitness

The low proportion of *A. cerana* worker brood successfully developing significantly reduced the reproductive success of the invasive lineage of *V. destructor*. As development anomalies occurred after the triggering of oogenesis of the foundress mites, earlier offspring stages were not affected. It is especially the number of older offspring stages that was underrepresented in *A. cerana*, with only 5% of the experimental infestations yielding viable daughters (Figure 3). We here confirm the occurrence of high brood susceptibility and its absence in Chinese populations of *A. cerana* and *A. mellifera*, respectively (Page et al., 2016). The occurrence of this trait in the original host population of the *V. destructor* Korean mite haplotype supports the idea that brood susceptibility is a general resistance mechanism of *A. cerana* against infestations by the invasive lineage.

### 4.3 Abnormal development of infested worker brood could trigger social immunity mechanisms

The previously reported ability of *A. cerana* to detect and remove large numbers of *V. destructor* from capped cells within minutes to 24 hr (Peng, Fang, Xu, & Ge, 1987; Rath & Drescher, 1990) was not confirmed by our results. In our study, *A. cerana* workers removed brood primarily between the second and the seventh day after mite infestation, in line with the observations of Rosenkranz et al. (1993) and Boot et al. (1999). With a peak before pupal stage, the frequency distribution of developmental stages at which infested Chinese *A. cerana* brood appeared to be arrested, corresponded to that observed in several Thai populations (Page et al., 2016). In both species, the temporal pattern of brood removal by adults corresponded very closely to the stages at which development seemed arrested following infestation (Figure 6). Our results support the idea of a causal link between infested brood degeneration and the initiation of social immunity based on the detection of abnormal development signals. A similar mechanism seems to occur in *A. mellifera* populations that possess the ability to remove *V. destructor* infested brood cells (a trait named *Varroa* sensitive hygiene; Mondet et al., 2016; Nazzi & Le Conte, 2016; Schöning et al., 2012). Whether the same factors and signals contribute to abnormal brood recognition in the two honeybee species remains to be investigated.

In our study, foundresses were missing from a proportion of experimentally infested cells. This was more frequently the case in the presence of adult *A. cerana* workers than in their absence, excluding an artifact of experimental infestation. This phenomenon could be due to a social immunity mechanism consisting in adult workers uncapping infested cells to remove *V. destructor* foundress mites and subsequently recapping these cells (Corrêa‐Marques & De Jong, 1998; Moretto, Guerra, & Bittencourt, 2006; Oddie, Dahle, & Neumann, 2017; Rath & Drescher, 1990). As a result of parasite departure or removal from its reproduction site, remaining brood developed normally and was not removed by adult workers. This finding again supports the idea that it is damage to the host rather than detection of the parasite itself that triggers hygienic behavior (Nazzi & Le Conte, 2016; Schöning et al., 2012).

When infestations over the whole experimental period did not impair the development of *A. cerana* workers, there were not removed and the mite could reproduce. Normal development and persistence until 1 day prior host emergence despite infestation occurred in five out of 60 cases. Three of the foundresses occupying these brood cells produced presumably mated daughters (Figure 5) and would thus have reproduced successfully upon host emergence. The Korean invasive lineage of *V. destructor* thus has the capacity to infest and successfully reproduce in *A. cerana* worker brood cells. At which frequency this phenomenon occurs naturally remains to be determined. Reproduction in worker brood has been previously observed in Vietnamese and South Korean honeybee populations (Boot et al., 1999; De Jong, 1988), but mite haplotypes could not be identified. It is thus unclear whether this ability is restricted to the invasive lineage of the Korean haplotype or if it is a trait common to all haplotypes of *V. destructor*. Determining the ability of other haplotypes to use worker brood for their reproduction will help understand whether this trait is instrumental for particular lineages (e.g., Korean and Japanese) of *V. destructor* to become invasive.

We did not investigate the fate of infested drone brood of *A. cerana*, but increasing infestation has been shown to hinder development and provoke the death of parasitized individuals that remain entombed in their cells (Boecking, Rosenkranz, & Sasaki, 1999; Rath, 1992, 1999). Host death is in this case conditional and depends on the degree of infestation of male individuals. The lethal reaction to infestation of both host sexes should lead to a decrease of the parasite's virulence and might have resulted in the co‐adaptation between *V. destructor* and *A. cerana*. Selecting for brood susceptibility in *A. mellifera* could thus accelerate the adaptation of mites to their new host and protect its populations sustainably.

### 4.4 Evolution of host susceptibility

Parasites may kill their hosts in many manners. They can directly affect host behavior and reduce host dietary intake (Goater & Ward, 1992), or indirectly affect its survival by enhancing susceptibility to predation (Combes, 1991; Holmes & Bethel, 1972), to other parasites (Price, 1980) or by triggering suicide (Smith Trail, 1980). We have previously proposed that the high susceptibility of *A. cerana* worker larvae to infestations with the invasive lineage of *V. destructor* is a form of suicide or social apoptosis that benefits the survival of the colony (Page et al., 2016). The physiological cause for high brood susceptibility to infestation by the invasive lineage of *V. destructor* needs to be elucidated to determine whether this trait originates from cellular apoptosis or from other mechanisms. For instance, repeating our experiment with mites of the Korean haplotype collected from *A. cerana* colonies, which are in general infected by fewer viruses (Yañez et al., 2016) is necessary to determine the potential role of the viruses in abnormal brood development (Mondet et al., 2016). Viruses generally occur at high loads in *A. mellifera* colonies and in the mites parasitizing them (Berthoud, Imdorf, Haueter, Radloff, & Neumann, 2010; Gisder, Aumeier, & Genersch, 2009; Tentcheva et al., 2004) and could disturb physiological processes in the brood (Francis, Nielsen, & Kryger, 2013; Highfield et al., 2009; Shen, Yang, Cox‐Foster, & Cui, 2005). Irrespectively of its proximate mechanisms, brood susceptibility can easily be maintained or self‐sacrifice can easily evolve in eusocial groups as the potential benefit acquired at the colony level from removing abnormal individuals to prevent future spread of pathogens and parasites can outweigh the costs at the individual bee level (Kralj & Fuchs, 2006; Page et al., 2016; Rueppell, Hayworth, & Ross, 2010; Smith Trail, 1980). Indeed, the high plasticity of social organization typical of honeybees allows for a rapid response of colonies to demographic changes, including losses of large proportions of its members that are rapidly compensated for (Shorter & Rueppell, 2012; Smith Trail, 1980).

## 5 CONCLUSION

Our previous findings in Thai *A. cerana* populations suggested that brood susceptibility prevents the spread in *A. cerana* of the invasive *V. destructor* lineage, now ubiquitous in Asia where *A. mellifera* is exploited (Page et al., 2016). The result described here shows that this phenomenon also occurs in the original host population of the Korean mite haplotype. Whether other haplotypes also induce developmental disturbance in *A. cerana* worker brood remains to be investigated. It is important to determine whether this phenomenon is a specific reaction to the invasive mite haplotype or if infestations by any mite haplotype trigger it. Determining the reproductive potential of other *Varroa* spp. haplotypes and species and identifying the resistance mechanisms in different *A. cerana* host populations will help to identify the factors allowing for or preventing parasitism, and those determining the virulence and host specificity of this parasite. A better understanding of interactions in this system (Navajas et al., 2010; Oldroyd, 1999; Rueppell, Hayes, Warrit, & Smith, 2011; Warrit et al., 2006) will not only provide fundamental knowledge on co‐evolution between hosts and parasites, but also potentially contribute to mitigating the detrimental effect of the invasive haplotype of *V. destructor* by allowing the development of more sustainable mite control strategies.

## CONFLICT OF INTEREST

None declared.

## AUTHOR CONTRIBUTIONS

V.D., P.N., H.Z., and F.H. conceived and designed the study; H.Z., V.D., and F.H. conducted the research; Z.L., Y.Q., P.P., S.W., L.L., and Z.W. performed the experiments; Z.L., V.D., and Y.Q. analyzed the data; Z.L., V.D., P.N., and H.Z. wrote the manuscript with contributions from all authors.
