# Supplementary figures and images for: Reproduction of parasitic mites Varroa destructor in original and new honeybee hosts
Source: Ecol Evol. 2018 Jan 22;8(4):2135–45. doi: 10.1002/ece3.3802 (PMC5817142; doi:10.1002/ece3.3802)

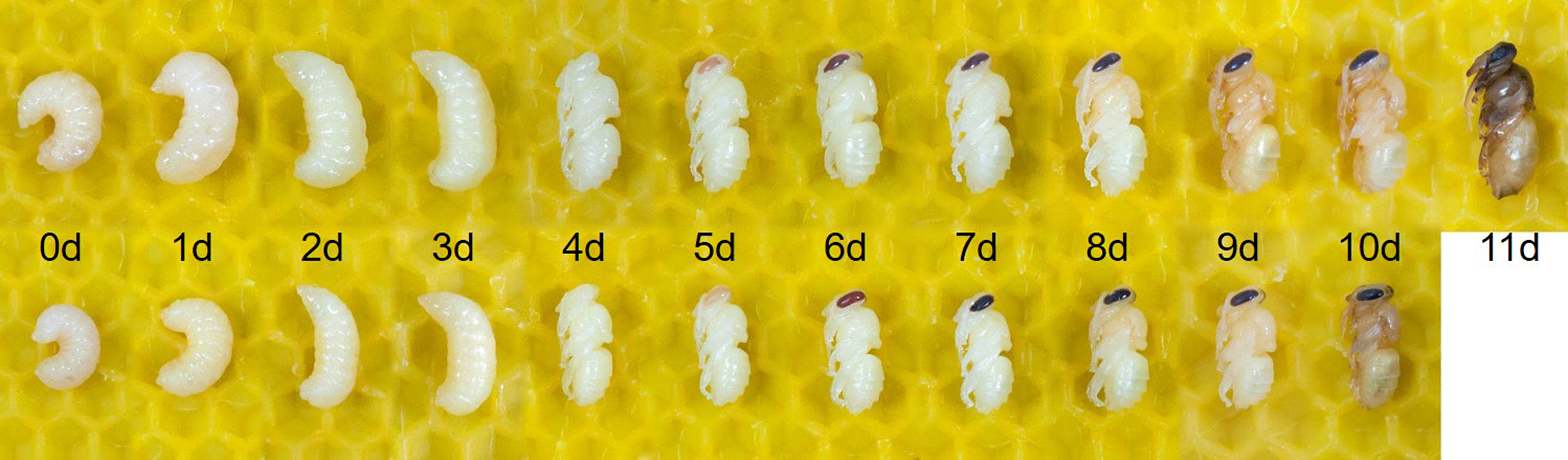

Supplement: Supplementary file 1 [file ECE3-8-2135-s001.tif]

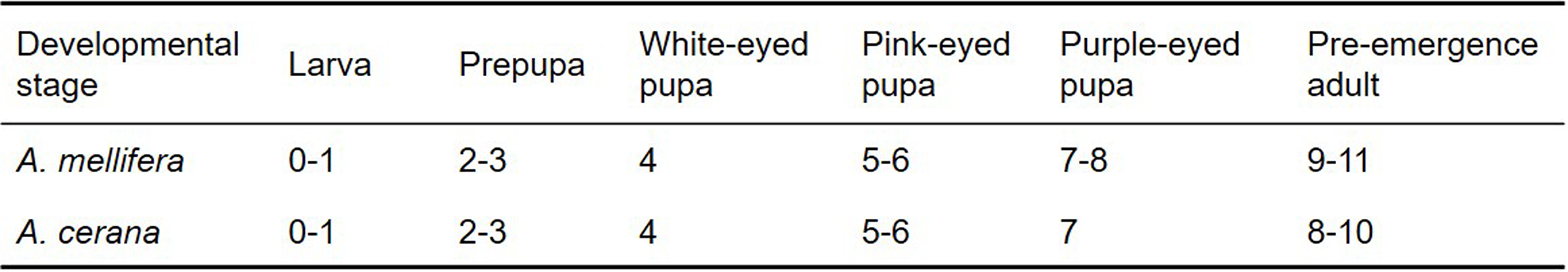

Supplement: Supplementary file 2 [file ECE3-8-2135-s002.tif]
