# Supplementary material for: Reproduction of parasitic mites Varroa destructor in original and new honeybee hosts
Source: Ecol Evol. 2018 Jan 22;8(4):2135–45. doi: 10.1002/ece3.3802 (PMC5817142; doi:10.1002/ece3.3802)
Supplement: Supplementary file 7 [file ECE3-8-2135-s007.docx]

**Table S1.** Uninfested worker brood development in *A. mellifera* and *A. cerana*. Duration of developmental stages is given in days after cell capping.

**Figure S1.** Appearance of normally developing worker brood of *A. mellifera* (above) and *A. cerana* (below) at one-day intervals. Day 0 corresponds to the day the cell was sealed with a wax capping by adult workers. Individuals were taken out of their cells for display.
